# Aging interventions get human

**DOI:** 10.18632/oncotarget.3173

**Published:** 2015-01-17

**Authors:** Brian K. Kennedy, Juniper K. Pennypacker

**Affiliations:** Buck Institute for Research on Aging, Novato, CA, USA

Over the last three decades, aging research has made great strides. At least in non-vertebrate animal models such as yeast and worms, it is possible to extend lifespan through reduced or ablated expression of hundreds of genes. The number of genes tested in mice are substantially less but the data so far is consistent with modulation of aging by numerous genes and pathways. More importantly, evidence exists that many of these genetic interventions extend healthspan and protect against the onset of age-associated chronic diseases. Recently, small molecules have entered center stage, with both natural products and clinically approved compounds reported to delay aging [1].

These findings raise the question of whether it is possible to forestall aging as an approach to maintain vitality and delay the onset of multiple chronic diseases simultaneously. However, there are significant hurdles to testing human aging drugs and many have been skeptical that aging interventions will ever enter the clinic. Among the foremost challenges, aging is not formally considered a disease by the FDA and the prospects of testing whether drugs extend human lifespan directly promises to be a long and exorbitantly expensive process. There is also the challenge of performing clinical trials in aging individuals who are still generally healthy. Foremost among these is the extra level of safety that will need to be incorporated since care must be taken not to do harm to healthy, older people. One potential solution is to test compounds against deleterious phenotypes associated with human aging - but which compound and which phenotype? This question has been debated extensively.

Sometimes the best approach is to start testing and let the results dictate the path forward. In this vein, Mannick et al. recently reported the results of the first human aging trial [2]. They chose a first generation derivative of the drug rapamycin (known as everolimus or RAD001), which has been shown to extend lifespan in all four major animal models of aging: yeast, worms, flies and mice [1]. Importantly, rapamycin, which is a direct inhibitor of the mTOR kinase, can extend lifespan by 25% in mice and even show efficacy when initiated in 20 month old mice [3, 4]. Most studies indicate that rapamycin extends healthspan as well [1]. Rapalogs, or rapamycin derivatives, are approved for treatment of several disease indications, but also have a range of side effects.

Mannick et al. chose to administer RAD001 to healthy people 65 and older over a six week period, followed by flu vaccine inoculation two weeks after suspending drug treatment [2]. Older individuals experience immunosenescence, characterized by an increased susceptibility to infection and a reduced response to vaccination. The goal of the study was twofold: to determine drug tolerability in the elderly and to see whether immunosenescence phenotypes were reversed. The use of a rapalog to improve immune function may seem paradoxical since one clinical use of this drug class is for immunosuppression after organ transplant. However, a six week regimen of rapamycin in elderly mice was shown to restore hematopoietic stem cell function, increase the response to influenza vaccination and lead to lifespan extension even though the drug was discontinued after this treatment window [5].

The findings from the Mannick study are encouraging. Recognizing that the side effects of RAD001 were most closely associated with trough levels of the drug, the authors chose three different dosing schemes designed to confer different levels of mTOR complex 1 inhibition while keeping trough levels minimized. Generally speaking the drug was well tolerated, with no evidence of an increase in adverse events at the two lower doses (0.5mg daily or 5mg weekly) and only a significant increase in mouth ulcers at the high dose (20mg weekly). Importantly, Mannick et al. found efficacy at both lower dose regimens of RAD001, demonstrating at least a 1.2 fold increase in the serologic hemagglutinin inhibition geometric mean titer ratio (HI GMT) of two of the three influence viruses represented in the vaccine at four weeks after inoculation [2]. This is a relevant target since prior studies have shown that a 20% increase in GMT ratio has been associated with reduced influenza illness [6]. Interestingly, RAD001 also appeared to broaden the serologic response, causing enhanced seroconversion to heterologous influenza strains not in the chosen influenza vaccine [2]. This finding is also suggestive of enhanced protection against influenza illness. The mechanisms behind this improved serologic response after rapamycin treatment require more studies but may involve an observed decrease in the percentage of PD-1 positive T cells, which accumulate with age and have a diminished antigen response. Unlike the study with rapamycin administration in aging mice [5], no increase in naïve lymphocytes was detected in humans [2].

It is intriguing that the benefits of RAD001 were apparent at the lower doses, which were only associated with partial mTORC1 inhibition. This may be consistent with recent findings in multiple tissues that mTORC1 activity increases with age, possibly driving age-associated pathologies and also that late administration of rapamycin in mice (20 months) confers enhanced lifespan at almost the same level as starting much earlier (9 months) [1]. Putting these findings together, the primary benefit of rapamycin and related rapalogs to aging may be through suppression of aberrant upregulation of the pathway that occurs during aging. This is encouraging, since lower dosing of RAD001 was not associated with an increase in adverse events.

Other clinically approved drugs have been linked to lifespan extension and protection against age-related diseases in animal models, including metformin and NSAIDs that have been prescribed millions of people. A recent retrospective examining patients with type 2 diabetes compared the effects on mortality rate of patients taking metformin or sulfonylurea monotherapy [7]. Not only did patients taking metformin have a lower mortality rate than those taking sulfonylurea, they had a lower mortality rate than other patients seeing the same doctor who did not have a diagnosis of metabolic syndrome. While there are caveats with any study of this nature, the findings suggest that metformin may be affecting basic aging processes that underlie multiple chronic disease and not just type II diabetes. One wonders whether many of the drugs used to treat early stage chronic disease may be effective at least in part because they target the biggest risk factor for these diseases: aging itself.

The study by Mannick et al. is groundbreaking but it sets the stage for testing drugs associated with delayed aging in healthy older human populations [2]. Whether rapalogs are the right drugs and immunosenescence is the right marker for healthspan remains to be determined, but it is critical for aging research to enter the clinic and this study is a fascinating initial foray. One hopes that it is the first of many, leading to successful interventions to extend human healthspan.
